# An Old Solution for a New Problem: Antiserum against Emerging Infectious Diseases

**DOI:** 10.3389/fpubh.2016.00178

**Published:** 2016-08-26

**Authors:** Victor Morais

**Affiliations:** ^1^Department of Biotechnology, Faculty of Medicine, Institute of Hygiene, University of the Republic, Montevideo, Uruguay

**Keywords:** antiserums, heterologous antibodies, infectious diseases, Ebola, snake venom

## Introduction

Heterologous neutralizing serums or antiserums consist of neutralizing antibodies produced mainly in horses or sheep and have been effectively used for more than a century. Antiserums were born in the golden age of microbiology when, in 1890, von Behering and Kitasato showed that the serum of a diphtheria-infected animal confers immunity against the same disease on naive animals (1, 2). Four years later, antiserum was used in humans. From that point, this method has always been demonstrated to be highly effective in the treatment of both infection and envenoming. However, antiserums did not have good outcomes with respect to safety in their initial applications, causing many life-threatening side reactions (3). Currently, in many applications, heterologous serums have been replaced by other drugs, such as antibiotics or homologous serums. However, in the case of envenoming from snakebites, scorpions and arachnids, antiserums remain the only effective treatment (4). In recent applications, antiserums have demonstrated a good safety profile, with <15% of patients having mild adverse reactions and <1% having severe reactions (4–6). The only weakness antiserums have is that, like most biological products, the induced reactivity in patients generates antibodies against the antiserum (7). This weakness causes the effectiveness and safety to be compromised in successive treatments, or, in other words, heterologous serums can only be used once.

## Heterologous Serums as Antimicrobials

In the first decades of the twentieth century, before the advent of antibiotics, heterologous serums were the best treatment choice against infectious diseases (8, 9). Many diseases were treated with heterologous serum with high effectiveness but with variable safety. For example, in 1904, a *Neisseria meningitidis* epidemic in New York City was controlled with a heterologous specific serum, decreasing the mortality by one-third (10).

Later in the twentieth century, antiserums began to be displaced by drugs with better safety profiles, antibiotics, and vaccination. However, for the treatment of envenomation, tetanus, diphtheria, and rabies, antiserums have seen continued successful use. Currently, the treatment for tetanus and diphtheria has been changed from antiserums to homologous serums obtained from healthy human donors, but in many countries, antiserums remain the only option for such treatment. In the case of snakebite and other envenomations, the antiserum is the only effective treatment.

## From Now Onward

For many emerging diseases, such as the Ebola virus, the risk–benefit equation for the use of antiserum appears to be highly tilted toward benefit. Additionally, it is necessary to use the antiserum only once because surviving patients demonstrate immunity after first contact. Another benefit is the low production cost, which makes this type of drug affordable for most countries (6, 11, 12). Unfortunately, while vaccination, monoclonal and homologous antibodies became the most popular solutions, antiserums had less success. Against many emerging diseases and future threats, however, antiserums could have a chance. Dixit and coworkers (6) are proposed antiserum as a possible solution to avian influenza, MERS-CoV, and viral hemorrhagic fevers. Heterologous serum also has the advantage that it can be made using recombinant proteins, which avoids the risk of manipulating the infective pathogen during the production stage.

## Conclusion

Antiserums are an old drug with more than a century of use. Perhaps for this, most pharmaceutical companies and scientist consider this type of drug as obsolete and opt for the new generation of antibodies (monoclonal, humanized).

However, antiserums still have some advantages to monoclonal antibodies, such as shorter product development time, and above all reduced costs in development and production. In summary, antiserums can be a good option for the treatment of emerging infectious diseases when other drugs are unavailable.

## Author Contributions

The author confirms being the sole contributor of this work and approved it for publication.

## Conflict of Interest Statement

The author declares that the research was conducted in the absence of any commercial or financial relationships that could be construed as a potential conflict of interest.

## References

[1] Chippaux JP Goyffon M. Venoms, antivenoms and immunotherapy. Toxicon (1998) 36:823–46. 10.1016/S0041-0101(97)00160-8 9663690

[2] Theakston RDG Warrell DA Griffiths E Report of a WHO workshop on the standardization and control of antivenoms. Toxicon (2003) 41:541–57. 10.1016/S0041-0101(02)00393-8 12676433

[3] Silverstein AM Clemens Freiherr von Pirquet: explaining immune complex disease in 1906. Nat Immunol (2000) 1:453–5. 10.1038/77874 11101860

[4] WHO. WHO Guidelines for the Production, Control and Regulation of Snake Antivenom Immunoglobulins. (2010). Available from: http://www.who.int/bloodproducts/snake_antivenoms/snakeantivenomguideline.pdf?ua=1 10.1051/jbio/2009043 20950580

[5] Otero-Patiño R Cardozo J Higashi H Núñez V Diaz A Toro M A randomized, blinded, comparative trial of one pepsin-digested and two whole IgG antivenoms for *Bothrops* snake bites in Uraba, Colombia. Am J Trop Med Hyg (1998) 58:183–9. 9580075 10.4269/ajtmh.1998.58.183

[6] Dixit R Herz J Dalton R Booy R. Benefits of using heterologous polyclonal antibodies and potential applications to new and undertreated infectious pathogens. Vaccine (2016) 34:1152–61. 10.1016/j.vaccine.2016.01.016 26802604 PMC7131169

[7] Morais V Berasain P Ifrán S Carreira S Tortorella MN Negrín A Humoral immune responses to venom and antivenom of patients bitten by *Bothrops* snakes. Toxicon (2012) 59:315–9. 10.1016/j.toxicon.2011.12.006 22206812

[8] Vital B Alguns casos de diphtheria tratados pelo serum anti-diphtherico. Rev Med São Paulo (1898) 1:51–6.

[9] Bull CG. The mechanism of the curative action of antipneumococcus serum. J Exp Med (1915) 22:457–64. 10.1084/jem.22.4.457 19867929 PMC2125359

[10] Berghman LR Abi-Ghanem D Waghela SD Ricke SC. Antibodies: an alternative for antibiotics? Poult Sci (2005) 84:660–6. 10.1093/ps/84.4.660 15844826 PMC7107177

[11] Kudoyarova-Zubavichene NM Sergeyev NN Chepurnov AA Netesov SV. Preparation and use of hyperimmune serum for prophylaxis and therapy of Ebola virus infections. J Infect Dis (1999) 179(Suppl 1):S218–23. 10.1086/514294 9988187

[12] Chippaux JP Boyer L Alagón A. Post-exposure treatment of Ebola virus using passive immunotherapy: proposal for a new strategy. J Venom Anim Toxins Incl Trop Dis (2015) 21:3. 10.1186/s40409-015-0003-1 25705218 PMC4336475

